# Extensive Broadband Near-Infrared Emissions from Ge_x_Si_1−x_ Alloys on Micro-Hole Patterned Si(001) Substrates

**DOI:** 10.3390/nano11102545

**Published:** 2021-09-28

**Authors:** Kun Peng, Ningning Zhang, Jiarui Zhang, Peizong Chen, Jia Yan, Changlin Zheng, Zuimin Jiang, Zhenyang Zhong

**Affiliations:** State Key Laboratory of Surface Physics, Department of Physics, Fudan University, Shanghai 200438, China; 15110190021@fudan.edu.cn (K.P.); 16110190037@fudan.edu.cn (N.Z.); 17110190029@fudan.edu.cn (J.Z.); 16110190016@fudan.edu.cn (P.C.); 19110190054@fudan.edu.cn (J.Y.); zcl@fudan.edu.cn (C.Z.); zmjiang@fudan.edu.cn (Z.J.)

**Keywords:** broadband near-infrared, ordered micro-holes, graded alloy, photoluminescence, guided resonant mode

## Abstract

Broadband near-infrared (NIR) luminescent materials have been continuously pursued as promising candidates for optoelectronic devices crucial for wide applications in night vision, environment monitoring, biological imaging, etc. Here, graded Ge_x_Si_1−x_ (x = 0.1–0.3) alloys are grown on micro-hole patterned Si(001) substrates. Barn-like islands and branch-like nanostructures appear at regions in-between micro-holes and the sidewalls of micro-holes, respectively. The former is driven by the efficient strain relation. The latter is induced by the dislocations originating from defects at sidewalls after etching. An extensive broadband photoluminescence (PL) spectrum is observed in the NIR wavelength range of 1200–2200 nm. Moreover, the integrated intensity of the PL can be enhanced by over six times in comparison with that from the reference sample on a flat substrate. Such an extensively broad and strong PL spectrum is attributed to the coupling between the emissions of GeSi alloys and the guided resonant modes in ordered micro-holes and the strain-enhanced decomposition of alloys during growth on the micro-hole patterned substrate. These results demonstrate that the graded Ge_x_Si_1−x_ alloys on micro-hole pattered Si substrates may have great potential for the development of innovative broadband NIR optoelectronic devices, particularly to realize entire systems on a Si chip.

## 1. Introduction

The near-infrared (NIR) spectroscopy is becoming an indispensable technique for a wide range of applications, such as night vision [1,2], surveillance [3], food analyses and environmental monitoring [4,5], biological imaging [6,7], and medical diagnostics [8,9]. The dominant principle of these applications is based on the unique absorption or scattering features related to the fundamental carbon-hydrogen (C-H) and oxygen-hydrogen (O-H) vibration modes, which generally cover a broad spectra range of ~1000–2000 nm in the NIR region (or so-called short-wavelength infrared) [4]. Accordingly, the broadband NIR light source and detector are highly in demand for simultaneous identification of various substances via the same photonic circuit. The traditional broadband NIR light sources are halogen lamps, high-pressure discharge lamps and super continuum lasers. They have disadvantages in spectra-stability, size, energy consumption and cost [9]. Many efforts have been devoted to realizing innovative broadband NIR light sources, such as light-emitting diodes based on unique phosphors [2,10] and PbS quantum dots in layered perovskite [11]. Considering the potential of monolithic optical-electronic integrated circuits on Si substrates to realize entire ‘systems on a chip’ [12], Si-based broadband NIR light sources and detectors are of great interest. In general, normal Si cannot emit or absorb light of wavelength longer than ~1.1 μm due to the limitation associated with the bandgap energy of ~1.12 eV. There have been several attempts to overcome this fundamental limitation. A hybrid structure of metal nanoantenna/Si has been proposed for the NIR detector by taking advantage of the direct light-harvesting and the energetic or “hot” electron generation during the plasma decay of nanoantenna [13]. The deep levels in Si formed via an ion implantation have been exploited to fabricate a waveguide-integrated infrared avalanche photodiode [14]. Dual Inversion Layers and Fowler−Nordheim Tunneling in a p-i-n junction (p-Si/AlOx/n-ZnO nanowire) have been employed to realize broadband photodetection [15]. Tensile-strained Si nanomembrane has been applied in photodetectors with an absorption limit of up to 1550 nm [16]. For longer wavelengths in mid-infrared range, GeSn alloy films on silicon substrates have also been studied due to the possible smaller direct bandgap [17]. More frequently, given the small bandgap of ~0.66 eV and the compatibility with the Si integration technology of Ge, Ge_x_Si_1−x_ (0 ≤ x ≤ 1) alloy promises to extend the operation wavelength in the NIR region [18,19,20,21,22,23]. Particularly, a stack of GeSi alloy quantum-wells (QWs) has been exploited to realize broadband NIR photodetectors in terms of the miniband formation due to the coupling of neighboring QWs or the composition of different energy transitions in QWs with different thicknesses and/or Ge contents [23]. Whereas the large lattice mismatch between Si and Ge restricts the number, the thickness and the Ge content of QWs to avoid strain-induced defects [24], their design and growth are quite complicated. In addition, charge tunneling to the smaller bandgap QWs can take place and, in turn, prevents equally efficient emission from all QWs. There is a well-known tradeoff between the wide spectral bandwidth and the degradation of performance of NIR optoelectronic devices based on GeSi alloys with higher Ge contents. So far, the broadband GeSi NIR light source or detector is still a significant challenge.

In this report, Ge_x_Si_1−x_ alloys with a graded increase of Ge content (x = 0.1, 0.15, 0.2, 0.25, 0.3) are grown on micro-hole patterned Si(001) substrates. The growth characteristics are disclosed. A strong and extensively broad photoluminescence (PL) spectrum is observed in the NIR wavelength range from 1200 nm to 2200 nm. This exhibits the broadest emissions from Ge_x_Si_1−x_ alloys and covers the widest NIR spectrum for practical applications. Detailed analyses of power- and temperature-dependent PL spectra, as well as the three-dimensional finite-difference time-domain (FDTD) simulations, provide an insight into the broad and strong PL spectrum. Our results demonstrate an innovative strategy of alloy growth on micro-hole patterned substrates to realize extraordinary broadband emissions. Given the rather broad and strong NIR emission and the compatibility with the Si integration technology, the graded Ge_x_Si_1−x_ alloys on micro-hole patterned Si substrates promise to be the superior candidate for a broadband NIR light source, particularly to realize entire systems on a Si chip.

## 2. Materials and Methods

**Figure 1:**
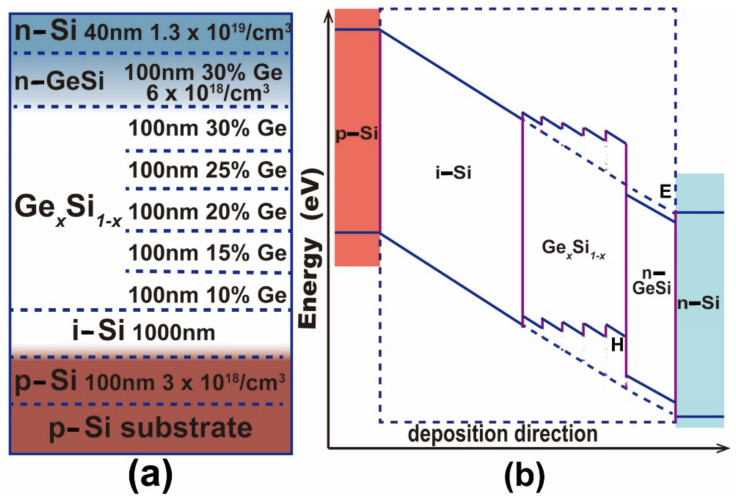
Schematic layer structure and energy band diagram. (**a**) The layer structure grown on the micro-hole patterned and the flat Si(001) substrates, (**b**) the energy band diagram of the sample.

The samples are grown on micro-hole patterned Si(001) substrates by solid source molecular beam epitaxy(MBE) in a Riber Eva-32 system. The ordered micro-holes in a hexagonal lattice on Si(001) (p-type, Boron doping) substrates are fabricated by a nanosphere lithograph [25]. The templates are cleaned by the RCA method with a subsequent HF dip to obtain a hydrogen-terminated surface. After a thermal desorption, a Si buffer layer of 100 nm with Boron (B) doping of ~3 × 10^18^ cm^−3^ is grown at a rate of 1Å s^−1^ at 500 °C. Then an intrinsic Si layer of 1μm is grown. Subsequently, a stack of five Ge_x_Si_1−x_ (x = 0.1, 0.15, 0.2, 0.25 and 0.3) alloy films is deposited at 480 °C. The thickness of each alloy film is 80 nm. The nominal compositions and the thicknesses of alloy films are determined by the growth rates of Ge and Si. The graded increase of Ge content with the largest value of 0.3 can effectively reduce misfit-induced defects. In addition, a Ge_0.3_Si_0.7_ alloy layer of 100 nm with phosphorus (P) doping of ~6 × 10^18^ cm^−3^ is grown at 430 °C. Finally, a Si capping layer of 40 nm with P-doping of ~1.3 × 10^19^ cm^−3^ is grown at 430 °C. The overall layer structure is schematically shown in Figure 1a. The same layer structure is also grown on a flat Si(001) substrate as a reference sample. Figure 1b schematically illustrates the corresponding energy band diagram of the reference sample. The B doping and the P doping naturally create a built-in electric field related to the p-i-n structure, which tilts the band alignment. This band alignment facilitates the accumulation of electrons and holes in regions E (around the interface between the n-GeSi and the n-Si) and H (intrinsic Ge_0.3_Si_0.7_ alloy layer), respectively, as shown in Figure 1b. The p-i-n structure also facilitates the realization of optoelectronic devices.

The surface morphology of the sample is characterized by scanning electron microscopy (SEM) (Zeiss Sigma) and atomic force microscopy (AFM) (Veeco DI Multimode V SPM) in tapping mode. The structure and composition of the sample are also investigated from a cross-sectional view using a field emission transmission electron microscope (TEM) (Thermo Scientific Talos F200i) operating at 200 kV. The electron-transparent TEM foil is prepared by focused ion beam (FIB) (Thermo Scientific Helios G4 CX). The Ge and Si distributions in the sidewalls of micro-holes are analyzed by X-ray energy dispersive spectroscopy (EDS) (Bruker Xflash 6T-30) attached to the microscope. PL measurements are performed in a closed-cycle helium cryostat with a temperature range of 17 to 300 K. The excitation source is a semiconductor laser (473 nm). The luminescence is analyzed by a monochromator (Omni-λ500, Zolix Instruments Co.) and detected with an extended InGaAs photodetector using the standard lock-in technique. The simulations of the emission spectra of Ge_x_Si_1−x_ alloys grown on micro-hole patterned Si (001) substrates are also carried out by the FDTD method. The boundary conditions of the perfectly matched layer are imposed in the *z* axis (out of plane). Periodic boundary conditions are employed in the x and y axes (in-plane). The emission intensity is obtained by integrating pointing vectors at the input port (*x*-*y* plane at *z* = 4.5 μm) in the time domain.

## 3. Results and Discussion

### 3.1. Morphologies and Structure Properties

**Figure 2:**
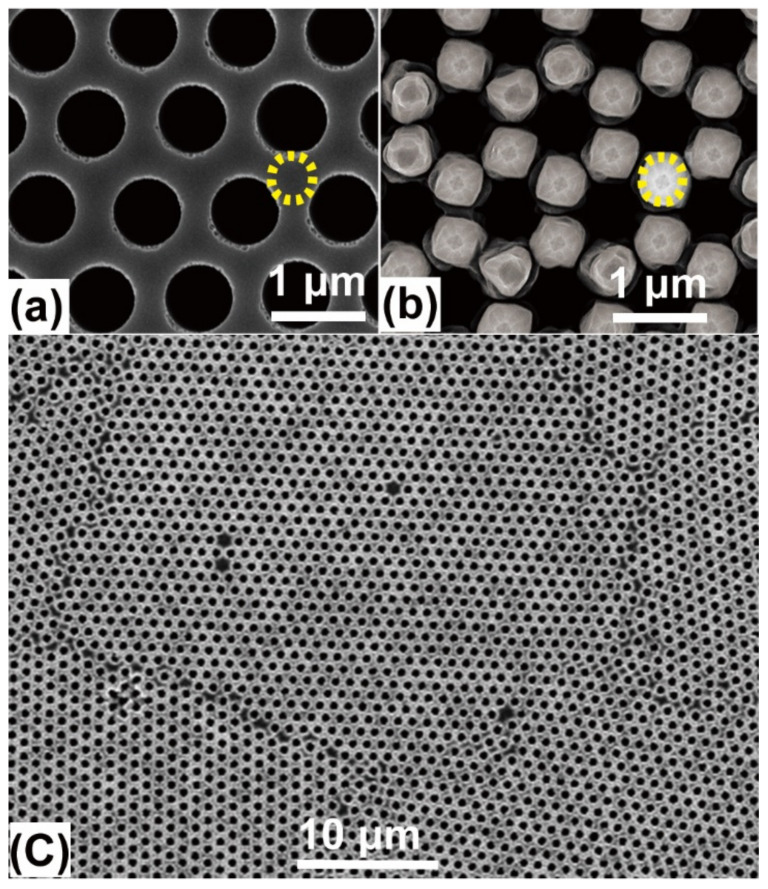
Top-view SEM images. (**a**) Ordered micro-holes on a Si(001) substrate, the micro-hole array after the growth of Si and graded Ge_x_Si_1−x_ alloy via MBE in (**b**) a small area, (**c**) a large area. The yellow dotted circles in (**a**,**b**) denote the regions in-between three micro-holes.

Figure 2a shows the typical top-view SEM image of ordered micro-holes on a Si(001) substrate. The micro-holes are arranged in a hexagonal lattice. The period, the diameter and the depth of micro-holes are about 1.0, 0.7 and 4.0 μm, respectively. These parameters can be readily modulated in the nanosphere lithograph [25]. Figure 2b shows the corresponding top-view SEM image of the micro-hole array after the growth of Si and graded Ge_x_Si_1−x_ alloys. The micro-holes remain with slightly reduced diameter of ~0.6 μm, which results from partial growth at their sidewalls. Moreover, barn-like islands with a base radius of ~260 nm and height of ~180 nm appear at the center regions in between the neighboring three micro-holes, as denoted by yellow dotted-circles in Figure 2a,b. This is consistent with the previous report on the low mismatch Ge_x_Si_1−x_ alloy islands on Si(100) substrates [26]. This barn-like island, with facets {1, 1, 1}, {1, 1, 3}, {15, 3, 23} and {1, 0, 5}, originates from the low surface tension and efficient strain relaxation of these facets [26]. The partial strain relaxation at the edges of the micro-holes facilitates the location of the barn-like island at the center region in-between the micro-holes, where misfit strain can be relaxed by the island formation. These microstructures are well ordered in a large area, as demonstrated in Figure 2c. Although some domain boundaries and point defects appear to degrade the long-range ordering of micro-holes, they can be considerably reduced by optimizing the nanosphere lithograph [25].

**Figure 3:**
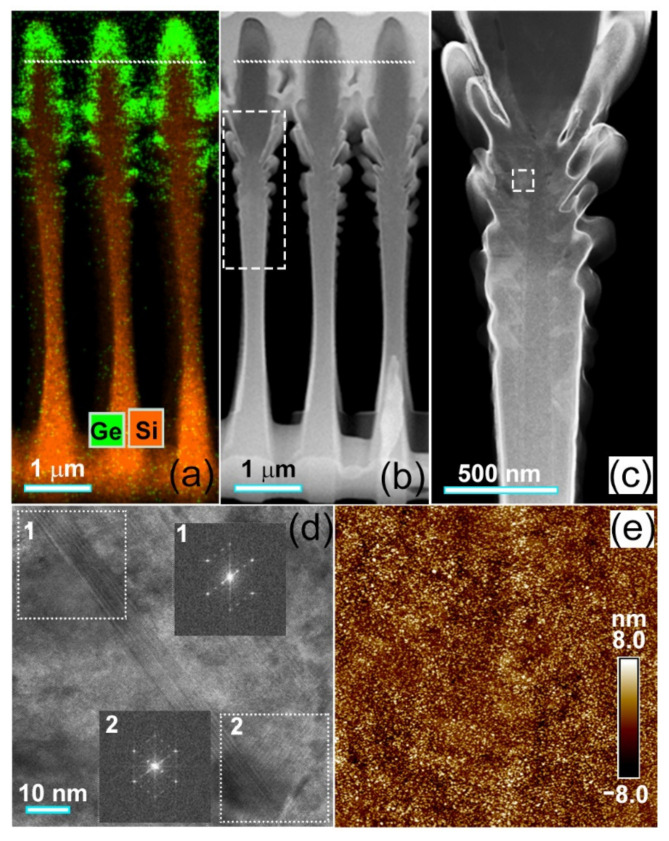
STEM and AFM images. (**a**) STEM-EDS mapping of Ge and Si in sidewalls, (**b**) STEM-HAADF image across the nearest neighboring micro-holes, (**c**) enlarged STEM-HAADF image of dash-boxed region in (**b**), (**d**) high resolution STEM image of dash-boxed region in (**c**), (**e**) AFM image (5 × 5 μm^2^) of the reference sample after growth on a flat substrate. The dotted line in (**a**,**b**) denotes the growth onset of Ge_x_Si_1−x_ alloys. The insets (1, 2) in (**d**) are the FT patterns of STEM images in the dot-boxes (1, 2).

Figure 3a shows the scanning transmission electron microscopy (STEM)-EDS mapping of Ge and Si in the sidewalls of micro-holes. Obviously, most Ge appears on the top of the sidewalls. Few Ge can be found at the bottom-half of the sidewalls. Figure 3b shows the corresponding high-angle annular dark field (HAADF) STEM image across the nearest neighboring micro-holes. Some branch-like nanostructures appear at the middle of the sidewalls of the micro-holes, considerably away from the growth onset of Ge_x_Si_1−x_ alloys denoted by dotted lines in Figure 3a,b. By careful inspection of Figure 3a,b, three main features can be seen. (i) The Si and GeSi alloys essentially grow on the upper-half of the sidewalls of micro-holes. Given the general incident flux of Si and Ge atoms tilted away from the axis direction of [001] of the micro-holes, the incident flux of Si and Ge atoms can hardly attach to the bottom part of the deep micro-holes, particularly after the formation of branch-like nanostructures at the sidewalls. (ii) The Ge-rich edge can be distinguished near the top of the sidewall. This demonstrates the segregation of Ge to the edge of the micro-holes during GeSi alloy growth. (iii) The branch-like nanostructures initiate during the Si growth at the sidewalls, since their root is mainly Si. These are induced by dislocations in the Si sidewalls, as shown in the enlarged STEM-HAADF image of Figure 3c. In the upper Si sidewalls with little dislocation, no branch-like nanostructure appears. We argue that these dislocations originate from the defects introduced during the long-time (over 20 min) plasma etching to obtain micro-holes with a depth of over 4 μm. These dislocations are mainly stacking faults, as demonstrated by the atomic resolution STEM-HAADF image (along the [110] zone axis) and the extra spots in the corresponding Fourier transformation (FT) patterns in Figure 3d. To learn about the evolution of those dislocations, further systematic studies are necessary. Considering the reduction of defects by the shorter etching time and the growth of GeSi alloy all over the sidewalls of the micro-holes, the depth of the micro-hole may be decreased. Figure 3e shows the AFM image of the surface morphology of the reference sample after the growth of Si and Ge_x_Si_1−x_ alloys on a flat substrate. The surface is quite smooth without a cross-hatch pattern [24], as it demonstrates few dislocations in the GeSi alloy layers. This result indicates that the graded increase of Ge content in GeSi alloy layers can efficiently suppress the formation of dislocations.

### 3.2. Power Dependent PL Spectra

**Figure 4:**
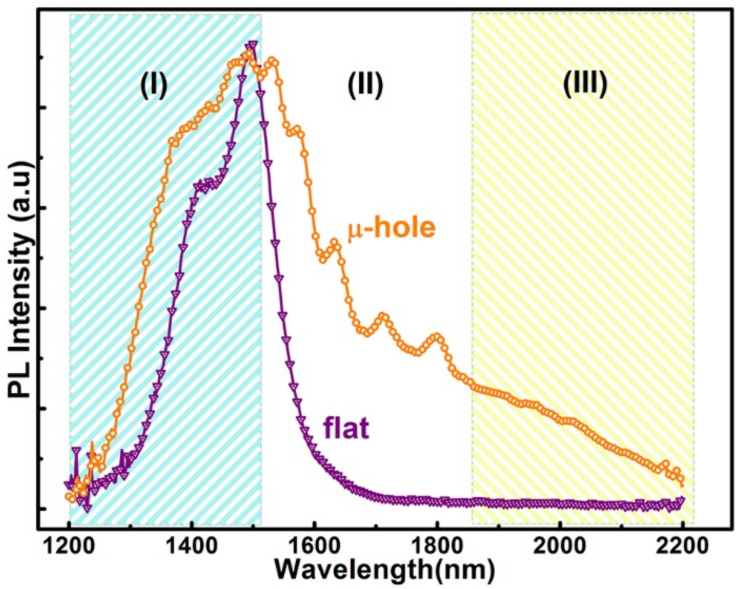
PL spectra. PL of graded Ge_x_Si_1−x_ alloys on the micro-hole patterned and the flat Si(001) substrates, denoted by ‘μ-hole’ and ‘flat’, respectively, at 17 K with the excitation power of 800 mW. (I), (II) and (III) denote three ranges of wavelength.

Figure 4 shows the PL spectra of graded Ge_x_Si_1−x_ alloys grown on the micro-hole patterned Si(001) substrate and the flat Si(001) substrate under the same growth conditions. Obviously, the PL spectrum of graded GeSi alloys on the micro-hole patterned substrate is substantially different from that of the reference sample on the flat substrate. Although there are five Ge_x_Si_1−x_ alloy layers with different Ge compositions (x = 0.1, 0.15, 0.2, 0.25, 0.3), the PL spectrum of the reference sample on the flat substrate is essentially composed of two peaks in the wavelength range of 1300–1600 nm, whereas the spectrum of the Ge_x_Si_1−x_ alloy on the micro-hole patterned substrate covers a rather broad wavelength range of 1200–2200 nm. Moreover, it can be separated into three regions. (I) In the range of 1200–1500 nm, the spectrum is similar to that of the reference sample on the flat substrate. (II) In the range of 1500–1850 nm, five satellite peaks can be distinguished. (III) In the range of 1850–2200 nm, a peak shoulder appears in the spectrum. Considering the longer wavelength and the unique peak features, the spectrum in regions (II) and (III) cannot be obtained directly from the graded Ge_x_Si_1−x_ alloys with x = 0.1–0.3.

**Figure 5:**
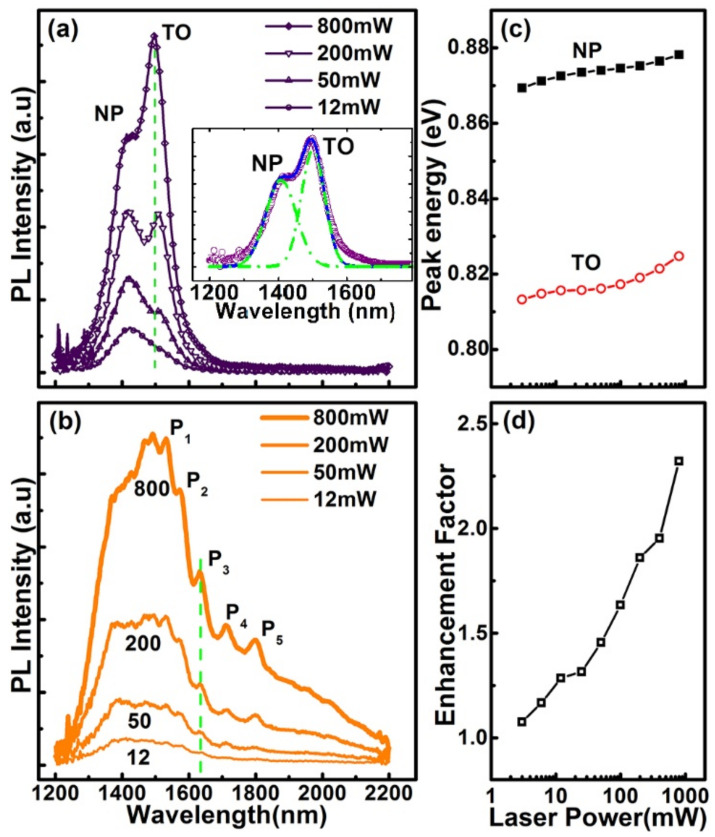
Power-dependent PL spectra. PL spectra of graded Ge_x_Si_1−x_ alloys (**a**) on the flat, (**b**) on the micro-hole patterned Si(001) substrate, (**c**) energies of peaks NP and TO in (**a**) as a function of excitation power, (**d**) the enhancement factor vs. the excitation power. The inset in (**b**) shows the fitting of the PL spectrum at 800 mW by two Gaussian peaks. The dashed green line in (**a**,**b**) is for eye guidance.

To clarify the origin of the extensive broadband spectrum, power-dependent PL spectra of the graded Ge_x_Si_1−x_ alloy layers on the flat and the micro-hole patterned substrate are obtained, as shown in Figure 5a,b, respectively. The spectra of the reference sample can be fitted by two Gaussian peaks around the wavelengths of 1408 nm and 1501 nm, as shown in the inset of Figure 5a. These two peaks are both blue-shifted with the excitation power, as shown in Figure 5c. This is the typical behavior of the type-II band alignment in GeSi/Si heterostructure due to band-filling and/or band-bending effects [27], whereas their energy difference of ~55 meV is nearly power-independent. These results demonstrate that these two PL peaks of the reference sample can be assigned as the non-phonon(NP) emission and its transverse-optical(TO) phonon replica of the GeSi alloy [27]. In addition, they mainly originate from the recombination of electrons and holes in regions E (around the interface between n-type Ge_0.3_Si_0.7_ and Si) and H (intrinsic Ge_0.3_Si_0.7_), as denoted in Figure 1b, which are the energetically favorable positions for electrons and holes, respectively. This means that the photon-generated carriers can efficiently drift to the energetically favorable positions around the Ge_x_Si_1−x_ alloy layer with the largest x value of 0.3 before their recombination. Emissions of the shorter wavelength from Ge_x_Si_1−x_ alloy layers with x < 0.3 can hardly be observed. This is consistent with the narrow electroluminescence spectrum of a quantum dot (QD) ensemble by charge tunneling to the smaller bandgap QDs [4]. This also suppresses the broadband emission. For PL spectra of the graded Ge_x_Si_1−x_ alloys on the micro-hole patterned substrate; the spectra in regions (I) of 1200–1500 nm and (III) of 1850–2200 nm are also slightly blue-shifted with the excitation power. Accordingly, they are dominated by the emissions of Ge_x_Si_1−x_ alloys but with x value different from 0.3, whereas, the positions of the satellite peaks in region (II) of 1500–1850 nm denoted by Pi (i = 1–5) are power-independent, as demonstrated in Figure 5b. This is distinctly different from the power-dependence of PL peaks of GeSi alloys [27]. Considering the ordered micro-holes in a hexagonal lattice, these satellite peaks are attributed to the coupling between the emissions of the graded Ge_x_Si_1−x_ alloys and the guided resonance modes in the ordered micro-holes [25]. Such a coupling not only effectively broadens the PL spectrum but also enhances the PL intensity. We can obtain the enhancement factor (EF) from EF = I_μ-hole_/I_flat_, where I_μ-hole_ and I_flat_ are the integrated intensity of PL peaks of graded GeSi alloys on the micro-hole patterned and the flat substrates, respectively. This is larger than one and remarkably increased with the excitation power, as shown in Figure 5d. The enhancement of PL spectra is generally attributed to the increased extraction efficiency of PL from the micro-structured surface and/or the reduced lifetime of excitation for the radiative recombination due to the coupling into the guided resonant modes of the ordered micro-holes. The former is essentially determined by the microstructure on the sample surface, which is power-independent. The present power-dependent EF demonstrates that the latter play the dominant role in the enhancement of PL spectra of the sample on a micro-hole patterned substrate. This result is consistent with the previous report on the enhanced PL spectra of SiGe coaxial quantum wells on ordered Si nanopillars [28].

### 3.3. FDTD Simulations

**Figure 6:**
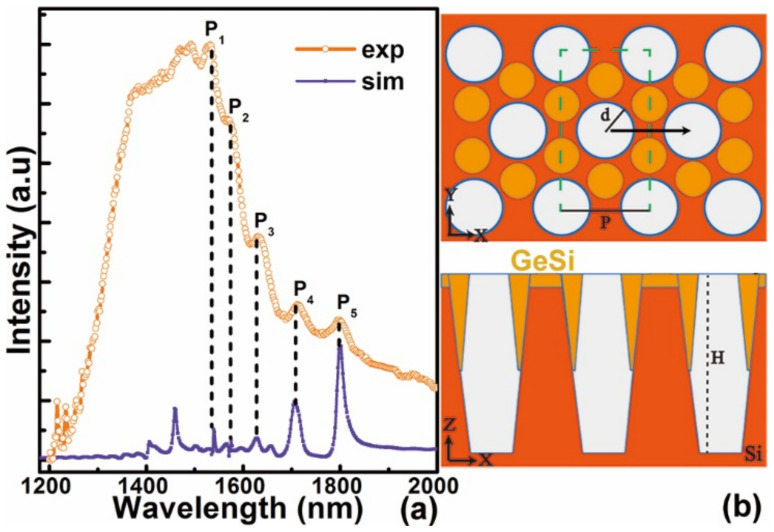
FDTD simulations. (**a**) Simulated spectrum of guided resonant modes of ordered micro-holes after graded Ge_x_Si_1−x_ (x = 0.1–0.3) alloys growth, as well as the experimental PL spectrum, (**b**) schematic illustration of the top view (up-panel) and the cross-sectional (along the black arrow in the up-panel) side-view (down-panel) of ordered micro-holes. The green dashed lines in the up-panel of (**b**) denotes the simulation cell of 1.02 μm × 1.77 μm × 11 μm. The mesh size in the FDTD simulation is 10 nm × 8.6 nm × 5 nm. The depth H of micro-hole is 5.8 μm. The period P is 1.02 μm.

The origin of the peaks Pi (i = 1–5) in region (II) of 1500–1850nm can be confirmed from the FDTD simulation of the emission spectrum of graded Ge_x_Si_1−x_ (x = 0.1–0.3) alloys grown on micro-hole patterned Si (001) substrates, as shown in Figure 6a. The geometrical profiles employed in the simulation are schematically shown in Figure 6b. Based on the SEM and the TEM images and the discussions above, the diameters at the top, the middle and the bottom of micro-holes are simplified to be 600 nm, 730 nm and 550 nm, respectively, as schematically demonstrated in the bottom-panel of Figure 6b. The barn-like morphologies at the regions in-between neighboring three micro-holes are also considered in the simulation. The simulated spectrum clearly demonstrates that there are guided resonant modes with the wavelengths well-matched with the experimental PL peaks Pi (i = 1–5) in region (II) of 1500–1850nm, whereas two additional peaks in the simulated spectrum with the shorter wavelengths (<1500 nm) are not as distinguishable in the experimental one. This may be attributed to the simple geometrical model used in the FDTD simulation, particularly without the consideration of the roughness at the sidewalls of the micro-holes.

### 3.4. Temperature Dependent PL Spectra

**Figure 7:**
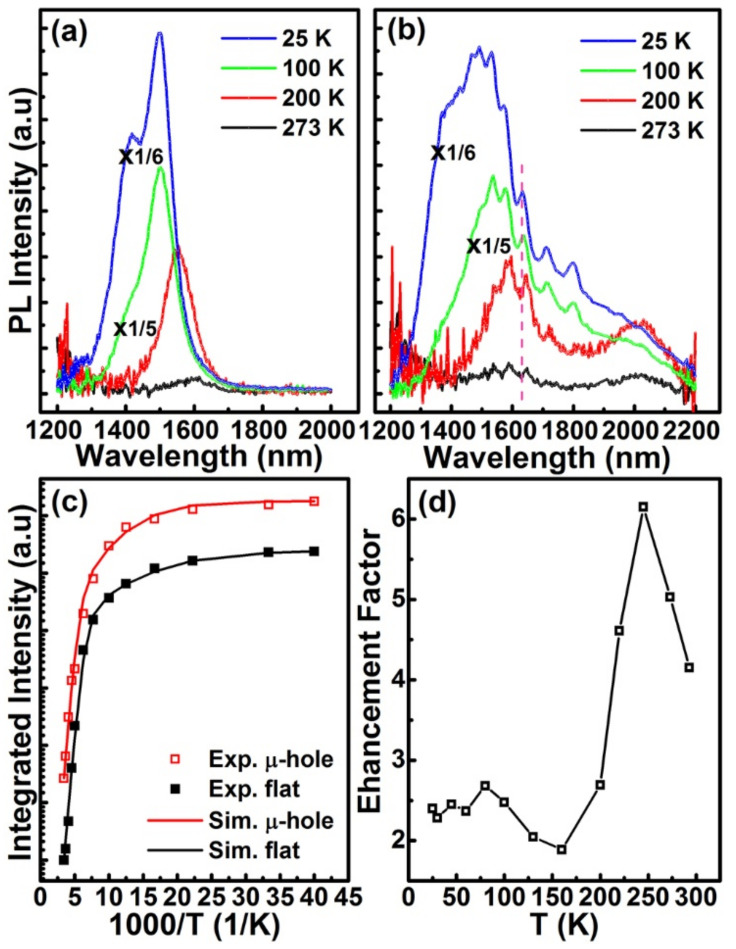
Temperature-dependent PL spectra. PL spectra of graded Ge_x_Si_1−x_ alloys (**a**) on the flat Si(001) substrate, (**b**) on the micro-hole patterned Si(001) substrate, (**c**) the integrated PL intensity vs. temperature, (**d**) the EF vs. temperature. For comparisons, the PL spectra at 25 K and 100 K in (**a**,**b**) are reduced by multiplying 1/6 and 1/5, as is denoted by ‘x1/6’ and ‘x1/5’, respectively. ‘Exp.’, ‘Sim.’, ‘μ-hole’ and ‘flat’ in (**c**) denote experiment, simulation, micro-hole patterned substrate and flat substrate, respectively. The dashed pink line is for eye-guide.

Figure 7a,b show the temperature-dependent PL spectra of graded Ge_x_Si_1−x_ alloy layers on the flat and the micro-hole patterned substrate, respectively. In both cases, a red-shift of PL peak with temperature is distinguished since the band gaps of both GeSi alloy and Si decrease with temperature. Even the peaks related to the guided resonant modes of the ordered micro-holes red-shift slightly with temperature. Such a red-shift is attributed to the variation of the refractive index of material with temperature. Surprisingly, along with the increase in temperature, a distinguished peak evolves from the former shoulder in region (III) of 1850–2200 nm in the PL spectra of the sample on the micro-hole patterned substrate, as demonstrated in Figure 7b. This is beyond the emission even of pure Ge. We argue that this peak mainly originates from Ge-rich domains in the graded Ge_x_Si_1−x_ alloys on the micro-hole patterned substrate. It has been found that segregation and/or spinodal decomposition are enhanced by strain during the step-flow growth of an alloy on a vicinal surface [29,30]. In addition, nonuniform surface chemical potential originated from the strain field and the vicinal surface energy biases the incorporations of the alloy components in different regions around the patterned surface. The vicinal surface and the nonuniform strain distribution naturally exist around the edge of the micro-hole. Accordingly, some Ge-rich domains can self-assemble around the edge of the micro-hole during the growth of graded Ge_x_Si_1−x_ alloys, particularly for x = 0.3, due to the accumulation of strain energy and the relative high Ge content [30]. This is confirmed by the TEM images in Figure 3. The rest in turn is Ge_0.3-δ_Si_0.7+δ_ alloy with a small δ that denotes the percentage of Ge separated into the Ge-rich domains. To obtain more details about the Ge-rich domain, further studies are necessary. Such a strain-dependent decomposition is also consistent with the previous report on non-uniform composition in GeSi islands [31]. The emissions of these Ge-rich domains dominate the spectrum in the long wavelength region (III) of 1850–2200 nm, particularly at high temperatures, since the higher Ge content provides deeper potential for holes. Similar emissions with smaller energy than the band gap of unstrained bulk Ge has been observed from Ge hut clusters on Si substrates due to the type-II band alignment and the misfit strain [32]. Given the limited number of Ge-rich domains, some photon-generated carriers can also radiatively recombine around Ge_0.3–δ_Si_0.7+δ_ alloys to dominate the spectra in the short wavelength region (I) of 1200–1500 nm. This scenario accounts for the slight blue-shift of the spectrum in region (I) of 1200–1500 nm in comparison with the PL peaks of the reference sample due to the small difference of Ge composition, as shown in Figure 4.

The formation of Ge-rich domains in the Ge_x_Si_1−x_ alloy grown on the micro-hole patterned substrate is also confirmed by the temperature-dependence of the integrated PL intensity, as shown in Figure 7c. Similar to the GeSi QDs, the integrated PL intensities of GeSi alloys as a function of temperature can be fitted by the following Equation (1) [33,34],

(1) $$I(T)=\frac{I(0)}{1+c_{1}\exp(-E_{1}/kT)+c_{2}\exp(-E_{2}/kT)}$$

where *I(0)* is approximated to be the integrated PL intensity at 17 K, *c*_1_ and *c*_2_ are the fitting parameters, *E*_1_ is the activation energy for the carriers to escape from the potential confinement of GeSi alloys, and *E*_2_ is the binding energy related to exciton dissociation. By fitting the experimental data in Figure 7c, the energies (*E*_1_, *E*_2_) for the samples on the flat and the micro--hole patterned substrates are (123 meV, 12.5 meV) and (132 meV, 19.8 meV), respectively. As shown in Figure 7b, the emissions in the long wavelength region (III) of 1850–2200 nm become more pronounced with the increase of temperature. The larger *E*_1_ demonstrates the deeper potential confinement, which corresponds to the larger Ge content, i.e., the Ge-rich domain. The larger *E*_2_ indicates the relatively small size of the Ge-rich domain [35]. Figure 7d shows the EF of PL intensity as a function of temperature. The light field distributions of guided resonant modes around the ordered micro-holes are not uniform and depend on the frequencies [25]. The strong coupling between the exciton emissions and the cavity modes of ordered micro-holes occurs mainly for excitons localizing at the light field maxima [36]. The variation of spatial distributions of excitons in the Ge_x_Si_1−x_ alloys on the micro-hole patterned substrate with the increase of temperature may affect the coupling between the emissions of Ge_x_Si_1−x_ alloys and the guided resonant modes. Accordingly, the EF is not monotonically changed with temperature, as shown in Figure 7d. The maximum EF is over 6 at 245 K. It is worth mentioning that the PL intensity of Ge_x_Si_1−x_ alloys on the micro-hole patterned substrate is reduced by defects in the sidewalls of micro-holes. By optimizing plasma etching processes to suppress defect formation in the Si sidewalls of micro-holes, the EF can be further increased. In addition, the cutoff wavelength of emissions will be shifted by applying an external voltage in the p-i-n structure due to a quantum confine Stark effect. However, such a shift is not so obvious for the general bias voltages [37]. By optimizing the parameters of micro-holes and the growth conditions (e.g., reducing Ge content), as well as taking into account the contributions of Si, the emission band can be extended further into the shorter NIR wavelengths.

## 4. Conclusions

In conclusion, a p-i-n structure with graded Ge_x_Si_1−x_ (x = 0.1, 0.15, 0.2, 0.25, 0.3) alloys is grown on micro-hole patterned Si(001) substrates. To relax misfit strain, barn-like islands self-assemble at the center regions in-between three neighboring micro-holes. Branch-like nanostructures appears at the sidewalls with many dislocations, which originate from the defects introduced during plasma etching. An extensive broadband PL spectrum is observed in the NIR range of 1200–2200 nm. It is attributed to the coupling between the emissions of Ge_x_Si_1−x_ alloy and the guided resonant modes in the ordered micro-holes, as well as the strain-enhanced decomposition of Ge_x_Si_1−x_ alloys during their growth on micro-hole patterned substrates. Moreover, the intensity of the PL spectra is remarkably enhanced, which can be over six times of that from the reference sample on the flat substrate at 245 K. Our results demonstrate an innovative strategy of growth of alloy on micro-hole patterned substrate to realize strong broadband spectrum. Considering the strong and rather broad wavelength range (1200–2200 nm) of emissions and the compatibility with the Si integration technology, the graded Ge_x_Si_1−x_ alloy layers on micro-hole patterned Si substrates will have a great potential in the broadband NIR light source particularly to realize entire systems on a Si chip.
